# Clinical characteristics and long-term outcome of CASPR2 antibody-associated autoimmune encephalitis in children

**DOI:** 10.1186/s13052-024-01727-5

**Published:** 2024-08-26

**Authors:** Donglei Liao, Saying Zhu, Lifen Yang, Ciliu Zhang, Fang He, Fei Yin, Jing Peng

**Affiliations:** 1 Department of Pediatrics, Xiangya Hospital of Central South University, Changsha, 410008 Hunan Province China; 2 Clinical Research Center for Children Neurodevelopmental disabilities of Hunan Province, Central South University, XiangyaHospital, Changsha, 410008 China

**Keywords:** Contactin-associated protein-2, Autoimmune encephalitis, Children, Long-term, Outcome

## Abstract

**Background:**

Contactin-associated protein-2(CASPR2) antibody-associated autoimmune encephalitis(AE) is rare in children. This study aimed to report the clinical characteristics and long-term outcome of CASPR2 autoimmunity in children to expand the disease spectrum.

**Methods:**

Children who were hospitalized in our hospital with clinically suspected AE from May 2015 to April 2022 and underwent neuronal surface antibodies detections were retrospectively analyzed. Clinical data of patients with CASPR2 autoimmunity were collected.

**Results:**

Patients who were positive for NMDAR-IgG, CASPR2-IgG, LGI1-IgG and IgLON5-IgG occupied 95.2%(119/125),3.2%(4/125),0.8%(1/125) and 0.8%(1/125), respectively.The median onset age of the 4 patients with CASPR2-IgG was 5.6 years. The most common symptoms were psychiatric symptoms/abnormal behavior(3/4) and sleep dysfunction(3/4). One patient developed a phenotype of Rasmussen encephalitis(RE). Tumor was absent in our patients. Two patients showed abnormal findings on initial brain magnetic resonance imaging(MRI) scans. All the patients showed favorable response to immunotherapy except the patient with RE experienced recurrent symptoms who finally achieved remission after surgery. All the patients had a favorable long-term outcome at the last follow-up(33-58months).

**Conclusions:**

CASPR2 autoimmunity may be the second most common anti-neuronal surface antibodies associated neurological disease in children. Psychiatric symptoms/abnormal behavior and sleep disorder were common in children with CASPR2-associated AE. Tumor was rare in those patients. Most pediatric patients had a favorable long-term outcome.

**Supplementary Information:**

The online version contains supplementary material available at 10.1186/s13052-024-01727-5.

## Indtroduction

Autoimmune diseases of the central nervous system (CNS) are composed of different disease entities with great heterogeneity [1]. Owing to the rapidly expanding repertoire of neural antibodies of the autoimmune diseases of the CNS, the spectrum of autoimmune CNS disorders has now been divided into antibody-associated disorders and those without defined antibodies such as multiple sclerosis, CNS vasculitis, Sydenham chorea, opsoclonus myoclonus ataxia syndrome, Behçet disease and Susac syndrome [2]. Anti-neural antibodies were classified as antibodies against the neuronal cell surface antigens, glial cell surface antigens and intracellular antigens, which were the biomarkers of autoimmune encephalitis(AE), antibody-associated demyelinating diseases and paraneoplastic neurologic syndromes, respectively [2]. Anti-N-methyl-D-aspartate receptor(NMDAR) encephalitis is the first described anti-neuronal surface antibody associated AE which was identified in 2007 [3], being the most common form of AE seen in children. Since then, a series of antibodies targeting neuronal surface antigens have been reported to be associated with AE. Anti-contactin-associated protein-2(CASPR2) antibody associated neurological disease is rare in adults, which is rarer in children. CASPR2 is a cellular adhesion molecule belonging to the neurexin family which is widely expressed in CNS and peripheral nervous system(PNS) [4]. Anti-CASPR2 antibody-associated neurological disease can manifest as autoimmune encephalitis, limbic encephalitis, peripheral nerve hyperexcitability syndrome, Morvan syndrome and cerebellar syndrome [5]. There were limited studies reported the anti-CASPR2 antibody-associated neurological diseases in pediatric patients. Herein, we aimed to summarize the clinical features and long-term outcome of children with CASPR2 antibody-associated AE from our center to expand the disease spectrum.

## Materials and methods

### Patients

Patients who were hospitalized in our pediatric department with possible AE met the criteria proposed by the Autoimmune Encephalitis International Working Group [6] from May 2015 to April 2022 underwent anti-NMDAR antibody analysis alone or neuronal surface antibodies panel detection including NMDAR-IgG, LGI1-IgG, CASPR2-IgG, GARBBAR-IgG, AMPAR-IgG and IgLON5-IgG. Paraneoplastic antibodies panel(anti-amphiphysin, -CV2, -PNMA2 (Ta), -Ri, -Yo, -Hu) was performed in all of the patients. Patients’ clinical data were recorded. The modified Rankin Scale (mRS) for children was used to assess the functional status of those patients [7]. It is a scale for measuring the degree of disability or dependence in the daily activities of patients. The mRS ranges from 0 to 6: no symptoms at all(score 0),no significant disabilities despite symptoms-behavior appropriate to age and normal further development(score 1), slight disability-unable to carry out all previous activities, but same independence as other age- and sex-matched children(score 2),moderate disability-requiring some help but able to walk without assistance(in younger patients adequate motor development despite mild functional impairment, reduction of 1 level on the gross motor function scale)(score 3), moderately severe disability-unable to walk without assistance(in younger patients reduction of at least 2 levels on the gross motor function scale)(score 4), severe disability-bedridden, requiring constant nursing care and attention(score 5),and dead(score 6) [7]. The mRS was evaluated during the acute phase, after immunotherapy in acute stage and at the last follow-up. Informed consent was obtained from each patient’s parents. And this study was approved by the Ethical Committee of Xiangya Hospital of Central South University.

### Antibody identification

The detection of NMDAR-IgG, LGI1-IgG, CASPR2-IgG, GABABR-IgG, AMPAR-IgG and IgLON5-IgG were performed by cell-based assays(Euroimmun, Germany or Guangzhou King Med Center for Clinical Laboratory). Paraneoplastic antibodies panel(anti-amphiphysin, -CV2, -PNMA2 (Ta), -Ri, -Yo, -Hu) was performed by using immunoblotting method (Euroimmun, Germany).

### Statistical analysis

Statistical analyses were conducted with SPSS version 26.0. Patients’ characteristics were summarized by expressing categorical variables as counts (proportions) and continuous variables as median and range.

## Results

### Clinical features of patients with definite antibody-positive AE

A total of 125 children with possible AE were identified with positive anti-neuronal surface antibodies. Ninety-seven out of 125 patients demonstrated anti-neuronal surface antibodies seropositive and 117 children with cerebral spinal fluid(CSF)-positive. The median onset age of 125 patients was 7 years(interquartile range(IQR) 4.65–10.1,range 0.4–15), with a male to female ratio of 54:71. The patients’ clinical features were shown in Table 1. The clinical manifestations of the 125 patients were as follows: psychiatric symptoms/abnormal behavior(99/125), movement disorders/dyskinesias(88/125), seizures(84/125), speech dysfunction(81/125), sleep disorders(76/125), decreased levels of consciousness(61/125),cognitive dysfunction/memory decline(41/125), autonomic dysfunction(39/125) and central hypoventilation(7/125). Three patients(2.4%,3/125) with anti-NMDAR encephalitis were identified with tumors(two with ovarian teratomas and one with primitive neuroectodermal tumor) and they all had removal of the tumors. The rate of abnormal initial brain magnetic resonance imaging(MRI) and electroencephalogram(EEG) was 56%(70/125) and 88.8%(111/125), respectively. All of the 125 patients received first-line immunotherapy: intravenous methylprednisolone(IVMP) pulse and/or intravenous immunoglobulins(IVIG) in the acute stage, and 8 out of 125 patients received plasma exchange. Forty-nine out of 125 patients were treated with second-line immunotherapies: rituximab(42/125), cyclophosphamide(16/125), mycophenolate mofetil (3/125), azathioprine(2/125), and tocilizumab(3/125). The median mRS max during acute phase was 4(range 1–6), and the median mRS after immunotherapy in acute stage decreased to 2(range 0–6). Ninety-nine out of 125 patients had follow-up information, and the median follow-up time was 21 months(IQR 8–40, range 0.3–137). The median mRS of last follow-up was 0(range 0–5), and 83.8%(83/99) had mRS ≤ 2 at last follow-up. Only one patient with anti-NMDAR encephalitis died in the acute phase in the current study. Seventeen out of 125(13.6%) patients experienced relapse.

**Table 1:** The clinical characteristics of patients with AE who had positive anti-neuronal surface antibodies

characteristics	patients with AE
onset age, y	0.4–15
sex(female), (%)	56.8%(71/125)
symptoms of AE,%	
psychiatric symptoms/abnormal behavior	79.2%(99/125)
movement disorders/dyskinesias	70.4%(88/125)
seizures	67.2%(84/125)
speech dysfunction	64.8%(81/125)
sleep disorders	60.8%(76/125)
decreased levels of consciousness	48.8%(61/125)
cognitive dysfunction/memory decline	32.8%(41/125)
autonomic dysfunction	31.2%(39/125)
central hypoventilation	5.6%(7/125)
tumors detection,%	2.4%(3/125)
abnormal initial brain MRI,%	56%(70/125)
abnormal initial EEG,%	88.8%(111/125)
treatments,%	
IVMP and/or IVIG	100%(125/125)
plasma exchange	8.4%(8/125)
rituximab	33.6%(42/125)
cyclophosphamide	12.8%(16/125)
mycophenolate mofetil	2.4%(3/125)
azathioprine	1.6%(2/125)
tocilizumab	2.4%(3/125)
mRS max, median, range	4,1–6(*n* = 125)
mRS after immunotherapy in acute stage, median, range	2,0–6(*n* = 125)
follow-up time, median, range(m)	21,0.3–137(*n* = 99)
mRS of last follow-up, median, range	0,0–5(*n* = 99)

AE: autoimmune encephalitis; EEG: electroencephalogram; IV: intravenous; IVIG: intravenous immunoglobulin; IVMP: intravenous methylprednisolone; m:months; MRI: magnetic resonance imaging; mRS: modified Rankin scale; y:year

Children with a diagnosis of anti-NMDAR encephalitis occupied 95.2%(119/125),based on clinical manifestations and detection of NMDAR-IgG in the CSF and/or serum; and the number of patients with anti-NMDAR encephalitis who showed myelin oligodendrocyte protein(MOG)-IgG seropositive and paraneoplastic antibodies seropositive was 12 and 7, respectively. The patients with CASPR2-IgG, LGI1-IgG and IgLON5-IgG seropositive occupied 3.2%(4/125),0.8%(1/125) and 0.8%(1/125), respectively.

### Clinical characteristics of patients with CASPR2 antibody-associated encephalitis

The median onset age of the 4 children with CASPR2-IgG seropositive was 5.6 years, ranging from 2.2 to 12 years. There were 3 females and 1 male. The patients’ clinical features were listed in Table 2. All the patients demonstrated anti-CASPR2 antibodies seropositive, with relatively low titers, with a median of 1:10, ranging from 1:10 − 1:30. Nobody had positive anti-CASPR2 antibodies tested in the CSF. And one patient had co-existence of MOG-IgG seropositive.Nobody exhibited with paraneoplastic antibodies seropositive. All of the 4 patients were previously healthy except case 3 was diagnosed with viral encephalitis at the age of 6 years. None of the patients had a positive family or past history of autoimmune disease.

**Table 2:** Clinical features of patients with CASPR2 antibody-associated autoimmune encephalitis

Case No.	case 1	case 2	case 3	case 4
Sex, onset age(y)	F,2.2	M,3.25	F,8y	F,12y
symptoms	fever, seizures, paralysis of the right limbs, irritability	fever, seizures, psychosis, abnormal behavior, abnormal postures, sleep dysfunction, unclear vision, limbs fatigue, regression of langauage: unable to communicate; regression of movement: unable to stand up and sit.	fever, psychiatric behavior, hallucination, paraesthesia, severe insomnia	psychosis, abnormal behavior, headache, conigtive decline, verbal and activity reduction, sleep dysfunction
CSF/Serum anti-CASPR2-ab	negative/positive(1:30)	negative/positive(1:10)	negative/positive(1:10)	negative/positive(1:10)
CSF/Serum anti-MOG-ab	Na/Na	negative/negative	Na/negative	negative/positive(1:10)
initial brain MRI	normal	widened bilateral cerebral sulcus and fissure, expanded supratentorial ventricle, mega cisterna magna	widened left ventricular temporal angle compared to contralateral side	normal
EEG	slow waves and sharp waves of the left hemisphere	generalized slowing	several EEG were perfomed, one was presented with epileptic discharge and others were normal	normal
initial CSF	WBC 0,protein 1.03 g/L	WBC 85,with a multinucleated cell predominance	unavailable	unavailable
immunotherapy	six times of IVIG, three times of IVMP, oral st taper and azathioprine	IVMP, oral st taper	three times of IVIG, two times of IVMP and oral st taper	IVIG
response to immunotherapy	prompt remission but with reccurent symptoms	prompt remission	slow remission	prompt remission
status of CASPR2-IgG during follow-up	persistent positive before surgey	converted to negative after 2 years from onset	converted to negative after 5months from onset	Na
mRS max	3	5	2	2
mRS at last follow-up	1	0	0	0
follow-up time	53 months	55 months	58 months	33 months
disease phenotype	Rasmussen encephalitis	autoimmune encephalitis	autoimmune encephalitis	autoimmune encephalitis

ab: antibody; CASPR2:contactin-associated protein-2;CSF: cerebral spinal fluid; EEG: electroencephalogram; F:female; IV: intravenous; IVIG: intravenous immunoglobulin; IVMP: intravenous methylprednisolone; M:male; MOG: myelin oligodendrocyte glycoprotein; MRI: magnetic resonance imaging; mRS modified Rankin scale; n.a.: not applicable; No.:number; st: steroids; WBC: white blood cell; y:year

The most common symptoms of the 4 patients with CASPR2 autoimmunity were psychiatric symptoms/abnormal behavior(3/4) and sleep dysfunction(3/4). Two patients developed seizures. Three patients demonstrated fever at disease onset but there were no specific pathogens associated with neurological symptoms. All of the 4 patients with CASPR2-IgG seropositive showed no peripheral nervous system involvement or autonomic symptoms. No tumor was detected in our patients. Initial brain MRI scans were normal in 2 and abnormal in 2 patients; one exhibited with widened bilateral cerebral sulcus and fissure, expanded supratentorial ventricle, mega cisterna magna; one showed widened left ventricular temporal angle compared to contralateral side. One patient (case 1) showed normal initial brain MRI but exhibited abnormal MRI results during follow-up. Two patients underwent lumbar puncture in local hospitals and the detailed results were unavailable. Another two patients demonstrated abnormal CSF findings; one showed elevated protein levels and the other showed mild pleocytosis with a multinucleated cell predominance.

EEG was performed in all of the 4 patients with CASPR2-IgG seropositive. One patient showed normal result and one patient underwent several EEG and showed epileptic discharge for once.The other two showed abnormal results; one showed slow waves and sharp waves of the left hemisphere and the other one showed slow background activity.

All of the 4 patients with CASPR2 antibody-associated encephalitis received first-line immunotherapy: IVMP pulse and/or IVIG, and only 1 patient(case 1) were added with second-line immunotherapy. The mRS max during acute stage ranged from 2 to 5 and the mRS after immunotherapy decreased to 1–2. One patient(case 1) relapsed. The median follow-up time was 54 months, ranging from 33 to 58 months after disease onset. All of the 4 patients had a favorable long-term outcome with an mRS score ≤ 1 at the last follow-up, one of whom received remission after hemisphere dissociation surgery and left hippocampal amygdala resection surgery. Three patients underwent anti-CASPR2 antibodies detection during follow-up; one patient showed persistent seropositive of anti-CASPR2 antibodies; another two subjects showed negative results of CASPR2-IgG when they performed detection again.

**Fig. 1:**
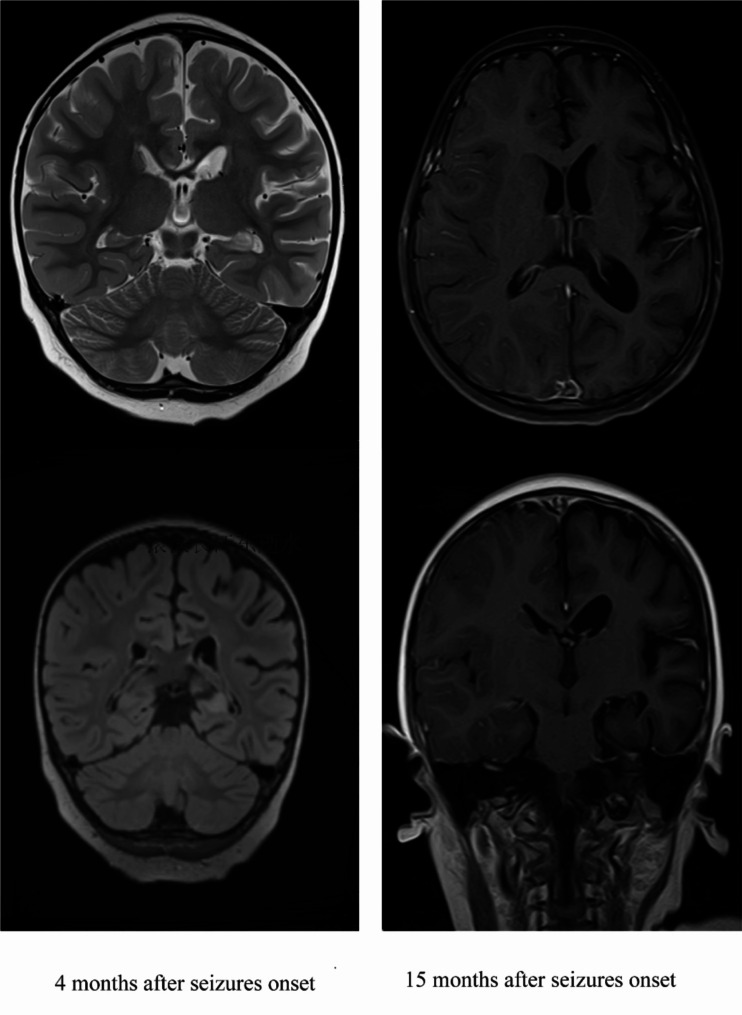
Brain MRI of case 1. MRI at 4 months after seizures onset showed smaller gyrus of the left frontal temporal parietal occipital lobe, with widening of cerebral fissure, slightly larger left ventricular temporal angle, smaller size and slightly T2/FLAIR hyperintensity signals of the left hippocampus. MRI at fifteen months after seizures onset showed atrophy of the left cerebral hemisphere and reduced lesion of the T2/FLAIR hyperintensity of the left hippocampus

In contrast to the other 3 patients with CASPR2 autoimmunity, case 1 showed no psychiatric symptoms or sleep dysfunction, and she showed persistent seropositive of CASPR2 antibodies and experienced relapse. Case 1 presented with focal seizures mainly on the right side, accompanied with disturbance of consciousness at the age of 2.2 years. She developed 2 times of fever which lasted 2–3 days 14 days and 7 days before onset of seizures, respectively. The frequency of seizures gradually increased and she developed status epilepticus for once. Thus, valproic acid and oxcarbazepine was added to her at our clinics but showed poor response. Trios-whole exome sequencing and mitochondrial genes showed negative results. Her initial MRI was normal. About 4 months after seizures onset, she developed irritability and paralysis of right-sided limbs. She couldn’t raise her right arm up and had difficulty in walking with her right leg (arm and leg Medical Research Council scale grade 4/5). Her MRI showed the gyrus of the left frontal temporal parietal occipital lobe is smaller than the contralateral side, with widening of cerebral fissure, the left ventricular temporal angle is slightly larger, the left hippocampus was smaller and slightly T2/fluid attenuation inversion recovery(FLAIR) hyperintensity signals were seen in the left hippocampus(Fig. 1). A suspicious diagnosis of Rasmussen encephalitis(RE) was established and she was hospitalized to perform brain biopsy and lumber puncture. The brain biopsy showed the deformation and atrophy of neuron and proliferation of glial cells. CASPR2-IgG was positive in serum with a titer of 1:30, but was negative in the CSF. Her symptoms were improved with IVIG and IVMP at the first hospitalization. But she still had focal seizures after discharge home and the frequency of seizures increased. She received 5 times of IVIG and 2 time of IVMP, and azathioprine was added to her in the following hospitalizations. She gained short seizures-free periods upon administration of immunotherapies but she remained paralysis of the ride limbs, with dragging steps of the right lower rimb when walking. She had anti-CASPR2 antibodies detected again at 9 months,12 months and 15 months after seizures onset, and the results remained positive. Fifteen months after seizures onset, her brain MRI showed atrophy of the left cerebral hemisphere and the lesion of the T2/FLAIR hyperintensity of the left hippocampus was reduced(Fig. 1), and hemisphere dissociation surgery and left hippocampal amygdala resection surgery were performed on her. She experienced several attacks within 1 month of surgery but she has achieved seizures free since then. Her muscle strength was improved but still showed mildly dragging steps when walking, and her mRS was 1 at the last follow-up.

## Discussion

The epidemiological data of anti-neuronal surface antibody associated autoimmune encephalitis are extremely limited and the frequency of CASPR2 autoimmunity remains obscure [8]. CASPR2 autoimmunity was the second most common type of anti-neuronal surface antibody autoimmunity in our cohort, which occupied 3.2% of known neuronal surface antibodies associated neurological disease and second only to anti-NMDAR encephalitis, similar to another two pediatric studies [1, 9].However, whether CASPR2 autoimmunity might be the second most common known anti-neuronal surface antibodies associated neurological disease in children warrants larger scale multi-center cohort study. It is mainly seen in males in adult patients and previous study showed that male children were more affected with this disease than females [9–11]. But a female predominance was seen in our study, which may be attributable to a small sample size. It is proposed that anti-CASPR2 antibodies mainly detected in serum rather than CSF [5, 9–11]. All of our patients had anti-CASPR2 antibodies detected in serum, nobody exhibited with CSF positive.

Psychiatric symptoms/abnormal behavior, movement disorders/dyskinesias, seizures and speech dysfunction were common in the total AE cohort in our study, in line with a recent summary of pediatric AE studies performed by the Autoimmune Encephalitis International Working Group [6]. Psychiatric symptoms/abnormal behavior(3/4) was also the most common symptom in patients with CASPR2 antibody-associated neurological disease in our study, which was in line with previous pediatric studies [9–11]. Seizures occurred in two of our patients with CASPR2 autoimmunity. A previous review of LGI1 and CASPR2 autoimmunity in children concluded that isolated epilepsy, epileptic encephalopathy or seizure disorder occupied 57.1%(8/14) in CASPR2-positive patients [12], and intractable epilepsy was also reported later in a multi-center study [9].A recent multi-center research has described the epileptic phenotypes in a population with autoimmune encephalitis, and the authors found multiple seizure types and prevalent involvement of temporal regions in patients with CASPR2 antibody-associated encephalitis [13]. Peripheral nerve hyperexcitability and Morvan syndrome were not observed in our patients and autonomic symptoms were also absent in our patients with CASPR2 associated encephalitis.

About 56% of patients with AE in our study exhibited abnormal initial brain MRI scans, which was a little higher than previously reported [6]. More than 50% adult patients with anti-CASPR2 associated syndrome exhibited abnormal brain MRI scans, which mainly involved medial temporal lobes and/or hippocampus [5]. Brain MRI scan in previous pediatric studies which enrolled less than 10 patients with CASPR2 autoimmunity reported that a minor part of patients showed variable abnormal findings;4 patients with T2 hyperintensity in the brain [10, 11, 14]; 2 with meningeal enhancement [15, 16]; 1 with brain atrophy [11]. A recent multiple-center study showed that 66.7% (10/15) children with CASPR2 antibody-associated autoimmune encephalitis revealed abnormalities of brain MRI which mainly located in cerebral cortex, thalamus, caudate nucleus, cerebral peduncle, white matter, hippocampus, globus pallidus and corpus callosum [9]. Two of our patients with CASPR2 antibody-associated encephalitis showed abnormal initial brain MRI scans; one exhibited with widened bilateral cerebral sulcus and fissure, expanded supratentorial ventricle, mega cisterna magna; one showed widened left ventricular temporal angle compared to contralateral side. Another patient had normal initial brain MRI but developed atrophy of the left cerebral hemisphere and T2/FLAIR hyperintensity of the left hippocampus during follow-up.

The rate of tumors detection was very low in our patients with AE. Nobody had tumor in our patients with CASPR2 autoimmunity, similar with most of the pediatric cases reported previously [10, 11, 14].Germinoma was discovered in one child who developed headache and hemiplegia of the left limb, which was considered to be associated with anti-CASPR2 antibodies [9]. Thymoma was the most common tumor in adult patients with anti-CASPR2 associated disease [5], which should also be taken into consideration in pediatric patients who underwent extensive tumor screening.

Most of the patients with AE in the current study showed favorable response to immunotherapies and had relatively good long-term outcome, with a low rate of relapse, in line with a recent study [1]. IVMP and/or IVIG were the most common managements and showed relatively favorable efficiency in our patients with CASPR2 antibody-associated encephalitis, consistent with most of the pediatric patients previously reported [9–11, 14].One patient diagnosed with Rasmussen encephalitis in our cohort was added with azathioprine due to recurrent symptoms. Only one patient with suspected RE associated with anti-CASPR2 antibodies relapsed in our study. Data of relapse in children with CASPR2 seropositive was limited. A recent study reported two out of 6 patients with anti-CASPR2 antibodies associated neurological autoimmunity relapsed [14].Wu et al. reported one out of 25 patients with CASPR2-related neurological syndrome relapsed, who showed anti-NMDAR antibodies positive in the CSF but negative results of anti-CASPR2 antibodies at relapse [9]. Thus, the relapse rate of anti-CASPR2 antibodies associated neurological disease might be relatively low. All of our patients with CASPR2 autoimmunity had a favorable long-term outcome at the last follow-up(33-53months).

One patient in our study was dual positive for CASPR2-IgG and MOG-IgG, which has not been reported in pediatric patients before, but was reported in an adult female recently [17]. The brain MRI of the adult patient in the literature showed multiple lesions scattered in brain, brainstem, and cervical and thoracic spinal cord, showing hypointensity on T1-weighted images, hyperintensity on T2-weighted and FLAIR images, with low-titer MOG-IgG in CSF(titer,1:1) and CASPR2-IgG in both serum and CSF (titers, 1:100 and 1:1), and a possible diagnosis of coexisting MOG-IgG-associated disease (MOGAD) and CASPR2 antibody-associated autoimmune encephalitis was established [17]. However, the patient in our cohort with dual positive antibodies had no abnormalities suggesting inflammatory demyelinating changes on brain MRI scan. Thus, we suggest that MOG-IgG may act as a secondary immune activation or represent a bystander effect in our patient. Besides, co-existence of anti-CASPR2 antibodies with other neuronal antibodies in patients with neurological diseases were not uncommon. LGI1-IgG, NMDAR-IgG, GABABR-IgG were reported to be identified in patients who harbored anti-CASPR2 antibodies in previous studies [5, 9, 11, 14]. LGI1 was the most common reported neuronal antibodies co-existing with anti-CASPR2 which might due to an overlap between peptides of LGI1 and CASPR2 [18]. Therefore, in patients with CASPR2 autoimmunity, tests of other neuronal antibodies should be performed to guide disease progression and prognosis.

One patient with anti-CASPR2 antibodies demonstrated a phenotype of RE, which has not previously been reported in pediatric patients with CASPR2 autoimmunity or RE. Antibodies targeted against neuronal antigens such as GluR3, ɑ-7 nicotinic acetylcholine receptor, GluR2-GluR3 subunit complex of the AMPAR, and mammalian uncoordinated (Munc)-18-1 have been discovered in a small portion of the RE patients [19, 20]. However, it was proposed that antigen-specific anti-neuronal autoimmunity is not the responsible players in RE and the immunopathogenesis underlying this disease remains obscure [19, 20]. But we observed that anti-CASPR2 antibodies in serum remained positive before surgery. Persistent seropositive of anti-CASPR2 antibodies in our patient suggested that this antibody might be involved in the disease or might implies a state of immune activation.

It was proposed that low titers of CASPR2-IgG posed a risk of false positive [21, 22]. But three out of 4 patients in our cohort exhibited monophase course and showed favorable responses to immunotherapies and the status of CASPR2-IgG in two of three patients converted to negative during follow-up, supporting that CASPR2-IgG may play a role in the diseases. Moreover, persistent seropositive of CASPR2-IgG might be involved in the disease process of the patient with suspected RE as mentioned above.

The strength of the current study was that we added the number of pediatric patients with CASPR2 autoimmunity in the literature and expanded the disease spectrum. The current study also had several limitations. Firstly, due to the retrospective design of the present study, we missed some important information of the patients. Secondly, the total sample of children with AE was small and derived from a single center. Thirdly, some of the patients in our cohort only underwent anti-NMDAR antibody detection and a panel of antibodies such as CASPR2, LGI1 and GABABR were not performed, which might lead to the missing of the phenomenon of co-existence of CASPR2-IgG with other anti-neuronal surface antibodies. Prospective large-scale multi-center study is warranted to investigate the frequency, clinical manifestations, imaging features, treatment responses and outcome of children with CASPR2 antibody-associated neurological disease.

## Conclusions

CASPR2 autoimmunity may be the second most common anti-neuronal surface antibodies associated neurological disease in children. Psychiatric symptoms/abnormal behavior was common in children with CASPR2 autoimmunity. Tumor was rare in children with anti-CASPR2 antibodies associated neurological disease. Pediatric patients with CASPR2 antibody-associated encephalitis might have a favorable long-term outcome.

### Electronic supplementary material

Below is the link to the electronic supplementary material.

Supplementary Material 1

Supplementary Material 2

## Data Availability

The datasets used during the current study are available from the corresponding author upon reasonable request.

## Abbreviations

AE: Autoimmune encephalitis
CASPR2: Contactin-associated protein-2
CNS: Central nervous system
CSF: Cerebral spinal fluid
EEG: Electroencephalogram
FLAIR: Fluid attenuation inversion recovery
IQR: Interquartile range
IVIG: Intravenous immunoglobulins
IVMP: Intravenous methylprednisolone pulse
MRI: Magnetic resonance imaging
mRS: Modified Rankin Scale
MOG: Myelin oligodendrocyte protein
NMDAR: N-methyl-D-aspartate receptor
RE: Rasmussen encephalitis
