# Supplementary material for: Clinical characteristics and long-term outcome of CASPR2 antibody-associated autoimmune encephalitis in children
Source: Ital J Pediatr. 2024 Aug 26;50:158. doi: 10.1186/s13052-024-01727-5 (PMC11346287; doi:10.1186/s13052-024-01727-5)
Supplement: Supplementary file 1 — Supplementary Material 1 [file 13052_2024_1727_MOESM1_ESM.docx]

Dear Raffaele,

We are very pleased to learn from your letter about the revision for our manuscript entitled " Clinical characteristics and long-term outcome of CASPR2 antibody-associated autoimmune encephalitis in children ". Thank you so much for your attention and the reviewer for his/her helpful comments and advice. We have revised the paper, and would like to re-submit it for your consideration. We have addressed the comments raised by the reviewer, and the amendments are highlighted in red in the revised manuscript. We hope that the revision is acceptable, and I look forward to hearing from you soon.

With best wishes,

Yours sincerely,

Jing Peng

My responses to the reviewer’s comments are below.

Reviewers' comments:

Reviewer #1:

1.Introduction- the abbreviations should be explained when used for the first time (AE for autoimmune encephalitis, CASPR2 for Contactin-associated protein-2).
Reply 1: Thank you for the advice. We had revised it in the introduction.

2. Introduction-the authors should better explain the background of Autoimmune diseases of the central nervous system, in particular antibody-mediated autoimmune encephalitis, highlighting the purpose of their study and why it is important for the current research.
Reply 2: Thank you for the suggestion. We have explained the background of the background of Autoimmune diseases of the central nervous system, in particular antibody-mediated autoimmune encephalitis: "Autoimmune diseases of the central nervous system (CNS) are composed of different disease entities with great heterogeneity. Owing to the rapidly expanding repertoire of neural antibodies of the autoimmune diseases of the CNS, the spectrum of autoimmune CNS disorders has now been divided into antibody-associated disorders and those without defined antibodies such as multiple sclerosis, CNS vasculitis, Sydenham chorea,opsoclonus myoclonus ataxia syndrome, Behçet disease and Susac syndrome. Anti-neural antibodies were classified as antibodies against the neuronal cell surface antigens, glial cell surface antigens and intracellular antigens, which were the biomarkers of autoimmune encephalitis(AE), antibody-associated demyelinating diseases and paraneoplastic neurologic syndromes, respectively. "

For highlighting the purpose of our study and why it is important for the current research, we have added in the introduction that: "There were limited studies reported the anti-CASPR2 antibody-associated neurological diseases in pediatric patients. "

3. Material and methods:
Patients
- Page 4, Line 34: the diagnostic criteria for autoimmune encephalitis should be explained or cited in references.
Reply 3: Thank you for the advice. We have added the diagnostic criteria for autoimmune encephalitis by citing a reference: Reference number 6.

4. - Page 4, Line 48: the modified Rankin Scale (mRS) should be explained (what is it, its scores etc..).Also, the information about when it is performed, should be included (T0, T1 or later).

Reply 4: Thanks for the suggestion. We have explained what is mRS and its scores definition in the Methods parts marked in red. And we have explained the mRS would be evaluated during the acute phase,after immunotherapy in acute stage and at the last follow-up.

5. Results:
- The authors should describe the total sample's characteristics (age, gender, clinical features, etc..). A table or graphic could be included.
- After that, the authors could proceed with the analysis of the patients with CASPR2 positivity, improving the discussion of their characteristics, even with the help of tables, figures or graphs, if necessary.
Reply 5: Thanks for the advice. We have summarized the total sample's characteristics including age, gender, clinical features, treatments and outcome. And we have also shown the total sample's characteristics in Table 1. And we proceed with the analysis of the patients with CASPR2 positivity after the total sample’s characteristics.

6. Explain the choice of the case 1's discussion, with similarities or differences with other three cases.
 Reply 6: Thanks for the suggestion. We have explained the choice of case 1’s discussion:In contrast to the other 3 patients with CASPR2 autoimmunity, case 1 showed no psychiatric symptoms or sleep dysfunction, and she showed persistent seropositive of CASPR2 antibodies and experienced relapse.

7. Discussion:
-also this section could be improved.
-In the discussion of the presence of seizures, a recent research has described the epileptic phenotypes in a population with autoimmune encephalitis (Matricardi S et al Epileptic phenotypes in autoimmune encephalitis: from acute symptomatic seizures to autoimmune-associated epilepsy. J Neurol Neurosurg Psychiatry. 2022). The authors found multiple seizure types and prevalent involvement of temporal regions.
Reply 7:Thanks for the suggestion. We have cited the suggested article and added it in the discussion of seizures: A recent multi-center research has described the epileptic phenotypes in a population with autoimmune encephalitis, and the authors found multiple seizure types and prevalent involvement of temporal regions in patients with CASPR2 antibody-associated encephalitis.

8. Limitation and streght of the study should be included and discussed.
Reply 8: Thank you for the advice. We have added the strength and limitations of our study:The strength of the current study was that we added the number of pediatric patients with CAPSR2 autoimmunity in the literature and expanded the disease spectrum. The current study also had several limitations. Firstly, due to the retrospective design of the present study, we missed some important information of the patients. Secondly, the total sample of children with AE was small and derived from a single center. Thirdly, some of the patients in our cohort only underwent anti-NMDAR antibody detection and a panel of antibodies such as CASPR2, LGI1 and GABABR were not performed, which might lead to the missing of the phenomenon of co-existence of CASPR2-IgG with other anti-neuronal surface antibodies.
