# Supplementary material for: Clinical characteristics and long-term outcome of CASPR2 antibody-associated autoimmune encephalitis in children
Source: Ital J Pediatr. 2024 Aug 26;50:158. doi: 10.1186/s13052-024-01727-5 (PMC11346287; doi:10.1186/s13052-024-01727-5)
Supplement: Supplementary file 2 — Supplementary Material 2 [file 13052_2024_1727_MOESM2_ESM.docx]

Cover letter

Dear editor,

We wish to submit the revised version of our article for publication in [Italian](https://www.sciencedirect.com/journal/clinica-chimica-acta) Journal of Pediatrics, titled ' Clinical characteristics and long-term outcome of CASPR2 antibody-associated autoimmune encephalitis in children'.

This study was approved by the Ethical Committee at the Xiangya Hospital of Central South University. Informed consent was obtained from all patients’ parents.

This manuscript has not been published or presented elsewhere in part or in entirety and is not under consideration by another journal. We have read and understood your journal’s policies, and we believe that neither the manuscript nor the study violates any of these. All the authors have read the manuscript and agreed to its being submitted for publication. All individuals listed as authors meet the appropriate authorship criteria, that nobody who qualifies for authorship has been omitted from the list, that contributors and their funding sources have been properly acknowledged, and that authors and contributors have approved the acknowledgement of their contributions. all author details on the revised version are correct, that all authors have agreed to authorship and order of authorship for this manuscript and that all authors have the appropriate permissions and rights to the reported data.

There are no conflicts of interest to declare.

Thank you for your consideration. We look forward to hearing from you.

Sincerely,

Jing Peng, M.D.& PhD

Director and Professor of Pediatrics Department,

Xiangya Hospital, Central South University

No.87,Xiangya Road, Changsha City, Hunan 410008, P.R. China

E-mail: ***pengjing@csu.edu.cn***
